# 5-Methyl­isoxazole-4-carboxylic acid

**DOI:** 10.1107/S1600536809048636

**Published:** 2009-11-21

**Authors:** De-Cai Wang, Wei Xu, Wen-Yuan Wu

**Affiliations:** a State Key Laboratory of Materials-Oriented Chemical Engineering, College of Pharmaceutical Sciences, Nanjing University of Technology, Xinmofan Road No. 5 Nanjing, Nanjing 210009, People’s Republic of China; b Department of Applied Chemistry, College of Science, Nanjing University of Technology, Xinmofan Road No. 5 Nanjing, Nanjing 210009, People’s Republic of China

## Abstract

In the title compound, C_5_H_5_NO_3_, the mol­ecule lies on a crystallographic mirror plane with one half-mol­ecule in the asymmetric unit. An intramolecular C—H⋯O inter­action is present. In the crystal, strong inter­molecular O—H⋯N hydrogen bonds result in the formation of a linear chain structure along [100], and there are also weak C—H⋯O hydrogen bonds between the chains which help to stabilize the crystal packing.

## Related literature

The title compound is an inter­mediate (Kotchekov *et al.*, 1985 ▶) for the synthesis of Leflunomide (Ree, 1998 ▶), an important anti­rheumatoid arthritis drug. For a related structure, see: Lee *et al.* (2002 ▶). 
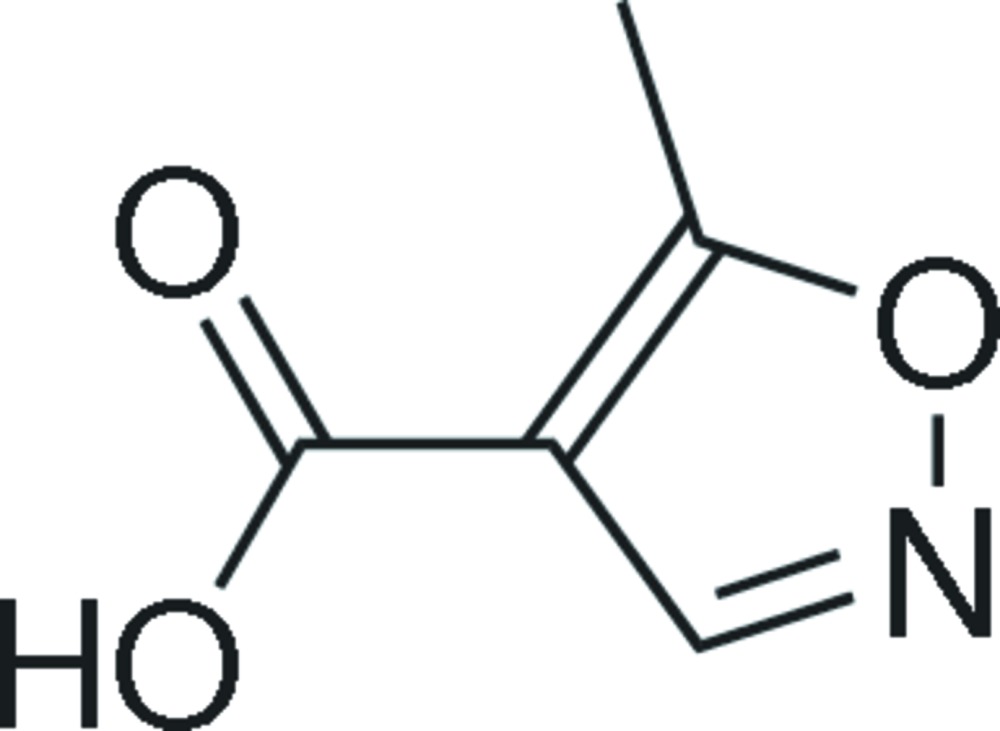


## Experimental

### 

#### Crystal data

C_5_H_5_NO_3_
*M* *_r_* = 127.10
Orthorhombic, 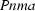

*a* = 7.2540 (15) Å
*b* = 6.4700 (13) Å
*c* = 12.273 (3) Å
*V* = 576.0 (2) Å^3^
*Z* = 4
Mo *K*α radiation
μ = 0.12 mm^−1^
*T* = 293 K
0.30 × 0.20 × 0.10 mm

#### Data collection

Enraf–Nonius CAD-4 diffractometer
Absorption correction: ψ scan (North *et al.*, 1968 ▶) *T*_min_ = 0.964, *T*_max_ = 0.988
1096 measured reflections
574 independent reflections
504 reflections with *I* > 2σ(*I*)
*R*_int_ = 0.042
3 standard reflections every 200 reflections intensity decay: none

#### Refinement

*R*[*F* ^2^ > 2σ(*F* ^2^)] = 0.040
*wR*(*F* ^2^) = 0.098
*S* = 1.00
574 reflections
63 parameters
H atoms treated by a mixture of independent and constrained refinement
Δρ_max_ = 0.26 e Å^−3^
Δρ_min_ = −0.16 e Å^−3^

### 

Data collection: *CAD-4 Software* (Enraf–Nonius, 1989 ▶); cell refinement: *CAD-4 Software*; data reduction: *XCAD4* (Harms & Wocadlo, 1995 ▶); program(s) used to solve structure: *SHELXTL* (Sheldrick, 2008 ▶); program(s) used to refine structure: *SHELXTL*; molecular graphics: *SHELXTL*; software used to prepare material for publication: *SHELXTL*.

## Supplementary Material

Crystal structure: contains datablocks global, I. DOI: 10.1107/S1600536809048636/fl2281sup1.cif

Structure factors: contains datablocks I. DOI: 10.1107/S1600536809048636/fl2281Isup2.hkl

Additional supplementary materials:  crystallographic information; 3D view; checkCIF report

## Figures and Tables

**Table 1:** Hydrogen-bond geometry (Å, °)

*D*—H⋯*A*	*D*—H	H⋯*A*	*D*⋯*A*	*D*—H⋯*A*
O1—H1⋯N1^i^	0.85	1.95	2.760 (3)	160
C4—H4⋯O2^ii^	0.93	2.33	3.217 (3)	159
C5—H3⋯O1	0.92 (2)	2.44 (5)	3.032 (4)	126 (5)

Symmetry codes: (i) 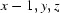; (ii) 
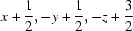
.

**Fig. 1.**
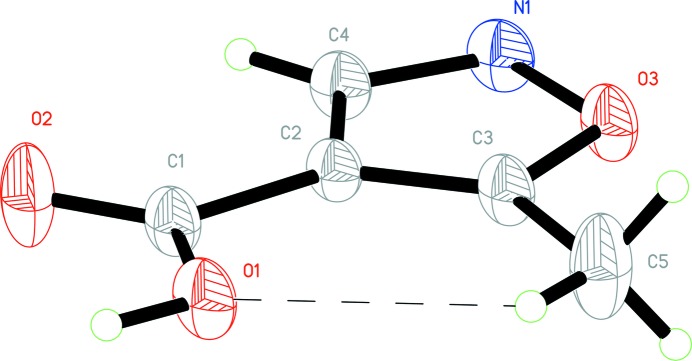
The molecular structure of (I), showing the atom-numbering scheme and displacement ellipsoids at the 30% probability level.

**Fig. 2.**
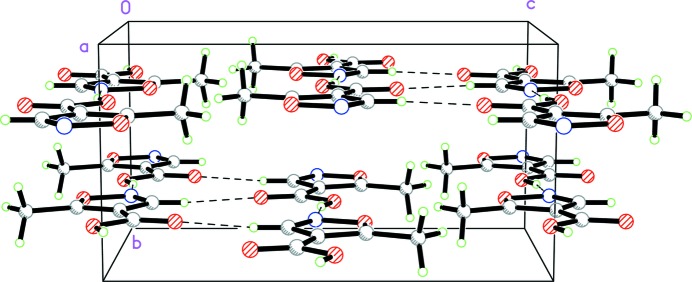
A packing diagram of (I). The intermolecular hydrogen bonds are shown as dashed lines.

## supplementary crystallographic information

### Comment

The title compound, (I), is a good organic intermediate (Kotchekov *et al.*, 1985) for the synthesis of Leflunomide (Ree, 1998), an important antirheumatoid arthritis drug. Here we report its crystal structure.

In the molecule of (I), (Fig. 1), the bond lengths and angles are within normal ranges. There is a mirror plane through all atoms except for two H atoms of the methyl group which are related by the mirror image - one above and one below the symmetry plane while the third methyly H atom lies in the mirror plane. An intramolecular C—H···O hydrogen bond helps to establish the molecular conformation. The molecule is similar to 3-Methylisoxazole-4-carboxylic acid methyl ester (Lee *et al.* (2002)).

Strong intermolecular hydrogen bonds are found between the H atom of carboxylic group and the N atom of the isoxazole ring (Table 1), which link the molecules into a one-dimensional supramolecular structure along the *a* axis. There are also weak C—H···O hydrogen bonds between adjacent two linear structures in the same symmetry plane (Fig. 2), which makes the linear structure two molecules wide.

### Experimental

A 500 ml solution of hydroxylamine hydrochloride (190 g, 2.7 mol) and sodium acetate trihydrate (370 g, 2.7 mol) was added to another 500 ml ethanol solution containing (*E*)-ethyl 2- (ethoxymethylene)-3-oxobutanoate. The mixture was stirred for 2 h and kept overnight at 273 K. Then the product of 5-methylisoxazole-4-carboxylate (II) (340–350 g) was extracted by dichloromethane (1200 ml).

(II) was then refluxed together with acetic acid (300 ml), water (300 ml), and concentrated HCl (300 ml) for 10 h, and crude sample of the title compound (260–270 g) was obtained. Pure compound (I) suitable for X-ray diffraction was collected by recrystallization from ethanol.

### Refinement

H atoms of methyl group were located in a difference map and refined freely. Then the other H atoms were positioned geometrically [O—H = 0.85 Å and C—H = 0.93 Å for aromatic H atoms] and constrained to ride on their parent atoms, with *U*_iso_(H) = *xU*_eq_(C,*O*), where*x* = 1.2 for aromatic and *x* = 1.5 for hydroxyl H atoms.

### Crystal data

**Table:**

C_5_H_5_NO_3_	*F*(000) = 264
*M**_r_* = 127.10	*D*_x_ = 1.466 Mg m^−^^3^
Orthorhombic, *P**n**m**a*	Mo *K*α radiation, λ = 0.71073 Å
Hall symbol: -P 2ac 2n	Cell parameters from 25 reflections
*a* = 7.2540 (15) Å	θ = 9–13°
*b* = 6.4700 (13) Å	µ = 0.12 mm^−^^1^
*c* = 12.273 (3) Å	*T* = 293 K
*V* = 576.0 (2) Å^3^	Block, colourless
*Z* = 4	0.30 × 0.20 × 0.10 mm

### Data collection

**Table:**

Enraf–Nonius CAD-4 diffractometer	504 reflections with *I* > 2σ(*I*)
Radiation source: fine-focus sealed tube	*R*_int_ = 0.042
graphite	θ_max_ = 25.3°, θ_min_ = 3.3°
ω/2θ scans	*h* = −8→8
Absorption correction: ψ scan (North *et al.*, 1968)	*k* = 0→7
*T*_min_ = 0.964, *T*_max_ = 0.988	*l* = 0→14
1096 measured reflections	3 standard reflections every 200 reflections
574 independent reflections	intensity decay: none

### Refinement

**Table:**

Refinement on *F*^2^	Secondary atom site location: difference Fourier map
Least-squares matrix: full	Hydrogen site location: inferred from neighbouring sites
*R*[*F*^2^ > 2σ(*F*^2^)] = 0.040	H atoms treated by a mixture of independent and constrained refinement
*wR*(*F*^2^) = 0.098	*w* = 1/[σ^2^(*F*_o_^2^) + (0.0214*P*)^2^ + 0.509*P*] where *P* = (*F*_o_^2^ + 2*F*_c_^2^)/3
*S* = 1.00	(Δ/σ)_max_ < 0.001
574 reflections	Δρ_max_ = 0.26 e Å^−^^3^
63 parameters	Δρ_min_ = −0.16 e Å^−^^3^
0 restraints	Extinction correction: *SHELXTL* (Sheldrick, 2008), Fc^*^=kFc[1+0.001xFc^2^λ^3^/sin(2θ)]^-1/4^
Primary atom site location: structure-invariant direct methods	Extinction coefficient: 0.108 (6)

### Special details

**Table:**

Geometry. All esds (except the esd in the dihedral angle between two l.s. planes) are estimated using the full covariance matrix. The cell esds are taken into account individually in the estimation of esds in distances, angles and torsion angles; correlations between esds in cell parameters are only used when they are defined by crystal symmetry. An approximate (isotropic) treatment of cell esds is used for estimating esds involving l.s. planes.
Refinement. Refinement of *F*^2^ against ALL reflections. The weighted *R*-factor *wR* and goodness of fit *S* are based on *F*^2^, conventional *R*-factors *R* are based on *F*, with *F* set to zero for negative *F*^2^. The threshold expression of *F*^2^ > σ(*F*^2^) is used only for calculating *R*-factors(gt) *etc*. and is not relevant to the choice of reflections for refinement. *R*-factors based on *F*^2^ are statistically about twice as large as those based on *F*, and *R*- factors based on ALL data will be even larger.

### Fractional atomic coordinates and isotropic or equivalent isotropic displacement parameters (Å^2^)

**Table:**

	*x*	*y*	*z*	*U*_iso_*/*U*_eq_	
C1	0.5625 (4)	0.2500	0.5646 (2)	0.0402 (7)	
C2	0.7524 (3)	0.2500	0.52268 (19)	0.0340 (7)	
C3	0.8152 (3)	0.2500	0.4188 (2)	0.0399 (7)	
C4	0.9135 (3)	0.2500	0.5867 (2)	0.0396 (7)	
H4	0.9130	0.2500	0.6625	0.048*	
C5	0.7282 (5)	0.2500	0.3096 (3)	0.0687 (12)	
H2	0.770 (4)	0.139 (4)	0.269 (2)	0.139 (15)*	
H3	0.601 (3)	0.2500	0.314 (4)	0.131 (19)*	
N1	1.0626 (3)	0.2500	0.52849 (19)	0.0442 (7)	
O1	0.4365 (2)	0.2500	0.48671 (16)	0.0493 (6)	
H1	0.3301	0.2500	0.5157	0.074*	
O2	0.5268 (3)	0.2500	0.65992 (16)	0.0684 (8)	
O3	1.0006 (2)	0.2500	0.41956 (14)	0.0446 (6)	

### Atomic displacement parameters (Å^2^)

**Table:**

	*U*^11^	*U*^22^	*U*^33^	*U*^12^	*U*^13^	*U*^23^
C1	0.0255 (14)	0.0539 (16)	0.0412 (15)	0.000	0.0037 (11)	0.000
C2	0.0212 (13)	0.0449 (14)	0.0359 (13)	0.000	−0.0005 (10)	0.000
C3	0.0203 (12)	0.0577 (18)	0.0416 (14)	0.000	0.0015 (11)	0.000
C4	0.0258 (13)	0.0530 (16)	0.0400 (14)	0.000	−0.0027 (11)	0.000
C5	0.0402 (18)	0.130 (4)	0.0357 (17)	0.000	−0.0017 (14)	0.000
N1	0.0224 (11)	0.0600 (16)	0.0503 (14)	0.000	−0.0049 (10)	0.000
O1	0.0183 (9)	0.0770 (15)	0.0525 (12)	0.000	0.0002 (8)	0.000
O2	0.0395 (12)	0.124 (2)	0.0412 (12)	0.000	0.0123 (9)	0.000
O3	0.0213 (10)	0.0677 (13)	0.0448 (11)	0.000	0.0048 (8)	0.000

### Geometric parameters (Å, °)

**Table:**

C1—O2	1.198 (3)	C4—N1	1.296 (3)
C1—O1	1.323 (3)	C4—H4	0.9300
C1—C2	1.470 (3)	C5—H2	0.923 (18)
C2—C3	1.353 (4)	C5—H3	0.92 (2)
C2—C4	1.408 (3)	N1—O3	1.410 (3)
C3—O3	1.345 (3)	O1—H1	0.8500
C3—C5	1.482 (4)		
			
O2—C1—O1	123.8 (3)	N1—C4—C2	112.7 (2)
O2—C1—C2	122.9 (2)	N1—C4—H4	123.7
O1—C1—C2	113.3 (2)	C2—C4—H4	123.7
C3—C2—C4	104.2 (2)	C3—C5—H2	110 (2)
C3—C2—C1	130.2 (2)	C3—C5—H3	112 (3)
C4—C2—C1	125.6 (2)	H2—C5—H3	111 (3)
O3—C3—C2	109.3 (2)	C4—N1—O3	104.8 (2)
O3—C3—C5	115.6 (2)	C1—O1—H1	108.9
C2—C3—C5	135.1 (2)	C3—O3—N1	108.97 (19)
			
O2—C1—C2—C3	180.0	C1—C2—C3—C5	0.0
O1—C1—C2—C3	0.0	C3—C2—C4—N1	0.0
O2—C1—C2—C4	0.0	C1—C2—C4—N1	180.0
O1—C1—C2—C4	180.0	C2—C4—N1—O3	0.0
C4—C2—C3—O3	0.0	C2—C3—O3—N1	0.0
C1—C2—C3—O3	180.0	C5—C3—O3—N1	180.0
C4—C2—C3—C5	180.0	C4—N1—O3—C3	0.0

### Hydrogen-bond geometry (Å, °)

**Table:**

*D*—H···*A*	*D*—H	H···*A*	*D*···*A*	*D*—H···*A*
O1—H1···N1^i^	0.85	1.95	2.760 (3)	160
C4—H4···O2^ii^	0.93	2.33	3.217 (3)	159
C5—H3···O1	0.92 (2)	2.44 (5)	3.032 (4)	126 (5)

Symmetry codes: (i) *x*−1, *y*, *z*; (ii) *x*+1/2, −*y*+1/2, −*z*+3/2.

## References

[bb1] Enraf–Nonius (1989). *CAD-4 Software*. Enraf–Nonius, Delft, The Netherlands.

[bb2] Harms, K. & Wocadlo, S. (1995). *XCAD4*. University of Marburg, Germany.

[bb3] Kotchekov, N. K., Khomutova, E. D. & Bazilevskii, M. W. (1985). *Zh. Ohshch. Khim.* **28**, 2736–2745.

[bb4] Lee, C. K. Y., Easton, C. J., Gebara-Coghlan, M., Random, L., Scott, A. P., Simpson, G. W. & Willis, A. C. (2002). *J. Chem. Soc. Perkin Trans. 2*, p. 2031–2038.

[bb5] North, A. C. T., Phillips, D. C. & Mathews, F. S. (1968). *Acta Cryst.* A**24**, 351–359.

[bb6] Ree, N. N. (1998). *Drugs Future*, **23**, 827–837.

[bb7] Sheldrick, G. M. (2008). *Acta Cryst* A**64**, 112–122. 10.1107/S0108767307043930 18156677

